# The Recent De Novo Origin of Protein C-Termini

**DOI:** 10.1093/gbe/evv098

**Published:** 2015-05-21

**Authors:** Matthew E. Andreatta, Joshua A. Levine, Scott G. Foy, Lynette D. Guzman, Luke J. Kosinski, Matthew H.J. Cordes, Joanna Masel

**Affiliations:** ^1^Department of Ecology & Evolutionary Biology, University of Arizona; ^2^Biochemistry and Molecular & Cellular Biology Graduate Program, University of Arizona; ^3^Department of Chemistry & Biochemistry, University of Arizona; ^4^Present address: Aegis Sciences, Nashville, TN; ^5^Present address: Program in Mathematics Education, Michigan State University, MI

**Keywords:** gene birth, stop codon readthrough, origin of novelty, protein structure

## Abstract

Protein-coding sequences can arise either from duplication and divergence of existing sequences, or de novo from noncoding DNA. Unfortunately, recently evolved de novo genes can be hard to distinguish from false positives, making their study difficult. Here, we study a more tractable version of the process of conversion of noncoding sequence into coding: the co-option of short segments of noncoding sequence into the C-termini of existing proteins via the loss of a stop codon. Because we study recent additions to potentially old genes, we are able to apply a variety of stringent quality filters to our annotations of what is a true protein-coding gene, discarding the putative proteins of unknown function that are typical of recent fully de novo genes. We identify 54 examples of C-terminal extensions in *Saccharomyces* and 28 in *Drosophila*, all of them recent enough to still be polymorphic. We find one putative gene fusion that turns out, on close inspection, to be the product of replicated assembly errors, further highlighting the issue of false positives in the study of rare events. Four of the *Saccharomyces* C-terminal extensions (to ADH1, ARP8, TPM2, and PIS1) that survived our quality filters are predicted to lead to significant modification of a protein domain structure.

## Introduction

The origin of novelty is a fundamental question in evolution (Mayr 1960; Müller and Newman 2005; Wagner and Lynch 2010). Many “novel” protein-coding sequences are rapidly diverging copies of older protein-coding sequences, following either duplication within a species or duplication associated with horizontal transfer from a different species (Ohno 1970; Long et al. 2003). However, some protein-coding genes are novel in a more fundamental way, being derived from noncoding sequences (Levine et al. 2006; Begun et al. 2007; Chen et al. 2007; Cai et al. 2008; Zhou et al. 2008; Knowles and McLysaght 2009; Siepel 2009; Tay et al. 2009; Toll-Riera et al. 2009; Xiao et al. 2009; Li, Dong, et al. 2010; Li, Zhang, et al. 2010; Donoghue et al. 2011; Tautz and Domazet-Lošo 2011; Wilson and Masel 2011; Wu et al. 2011; Yang and Huang 2011; Ding et al. 2012; Murphy and McLysaght 2012; Xie et al. 2012; Long et al. 2013; Reinhardt et al. 2013; Suenaga et al. 2014; Zhao et al. 2014). Because de novo gene evolution is hard to detect, known cases may be the tip of the iceberg, and noncoding sequences may be a common source of orphan genes, that is, genes that lack detectable homology to known proteins outside a given lineage (Tautz and Domazet-Lošo 2011; Wu et al. 2011; Ruiz-Orera et al. 2014) This hypothesis is supported by the statistical tendency for young genes as a whole to show characteristics that are better explained by de novo origination than by gene-duplication-divergence, including short length, fewer exons, and fewer domains (Neme and Tautz 2013).

Conversion of noncoding sequences into coding-sequences also occurs in a more limited way involving only part of a gene, such as new or expanded coding exons (Nurminsky et al. 1998; Kondrashov and Koonin 2003; Sorek 2007; Lin et al. 2009) or incorporation of 3′-untranslated regions (UTRs; Giacomelli et al. 2007; Vakhrusheva et al. 2011) or 5′-UTRs (Wilder et al. 2009) into coding regions. These latter processes could lead to expansion or modification of existing protein domain structures, which can vary substantially in length (Sandhya et al. 2008, 2009). Instances of the co-option of only part of a gene might be more numerous than completely de novo coding genes; for example, 43 instances of 3′-UTR incorporation are known in *Saccharomyces* (Giacomelli et al. 2007), and another 13 are known in bacteria (Vakhrusheva et al. 2011).

To understand the evolutionary process of conversion of noncoding sequences to coding, it is helpful to have well-supported examples that are very recent, indeed not yet fixed. Liti et al. (2009) reported 134 subtraction polymorphisms in *Saccharomyces cerevisiae* and *Saccharomyces paradoxus*, mostly near C-termini and sometimes in essential genes, but did not describe these results in detail or report results on additions. Fitzpatrick et al. (2011) identified 376 examples of stop codon polymorphisms (SCPs), and reported these as subtractions. However, no outgroup was used, and some of these polymorphisms may be additions.

Here, we describe a more thorough analysis of SCPs in each of these two *Saccharomyces* species, using the other species as an outgroup in order to distinguish additions from subtractions. We find 54 examples of 3′-UTR incorporation alleles that have not yet become fixed, after applying stringent quality controls to avoid false positives. By “false positive,” we mean either that the change is observed only as a result of a sequencing or other technical error, or that the change is real but applies to an opening reading frame (ORF) that is not a true protein-coding gene. Our quality controls include the exclusion of singleton alleles as possible sequencing or other one-off errors, a reassessment of the protein-coding status of the annotated genes undergoing a C-terminal extension, and the exclusion of one gene fusion event as a likely assembly error. Because our examples of C-terminal extension are of very recent origin, they can shed light on the process and not merely the end point of conversion of noncoding sequences into coding. At least four among the 54 additions to C-termini are interesting from a protein structure perspective.

## Materials and Methods

### Yeast Data Sources

For each annotated gene in one of the two reference genomes, ORF plus UTR data for *S. cerevisiae* and *S. paradoxus* sequences were downloaded from the *Saccharomyces* Genome Database (SGD) (Cherry et al. 2012) using a release that was current as of May 20, 2011. SGD provides the *S. cerevisiae* reference genome. The *S. paradoxus* reference genomes were originally sequenced by Kellis et al. (2003) but include a number of substantial updates since first publication. Full genome sequences of 38 more *S. cerevisiae* and 35 more *S. paradoxus* strains were downloaded from the *Saccharomyces* Genome Resequencing Project (Liti et al. 2009) using a release that was current as of May 20, 2011. The sequenced yeast strains are either fresh environmental isolates or strains adapted to laboratory conditions over the longer term. In nature, *Saccharomyces* is found as a diploid whose high rate of selfing leads to little heterozygosity (Tsai et al. 2008).

### Sequence Selection and Alignment

Genes were excluded if they were marked dubious by SGD, had fewer than 150 nt of 3′-UTR sequence available, were nonchromosomal, lacked clearly annotated homology between the two yeast species, or were annotated as a “retrotransposon.” These exclusions reduced the number of genes, totaled across both species, from 11,368 to 10,922. BLAST hits to 600 nt at the 3′-end of each remaining reference coding sequence were found for each of the yeast strains (Altschul et al. 1997). After preliminary quality screening based on BLAST e-values and synteny, a reciprocal best hit was required to establish homology between the reference strain and a second strain of the same yeast species. This left 398,114 sequences spread across 10,725 genes.

For each gene, sequences for each strain of that species, including the reference, were aligned using MUSCLE (Edgar 2004). Alignment can be distorted by gaps at the outer edges, so we used an iterative algorithm to extend and prune sequences until the alignment edges were free of gaps. Alignment began with the ORF plus 150 nt of the 3′-UTR. Sometimes, after extension and pruning to obtain gap-free alignment edges, not all sequences contained a stop codon in-frame with the annotated start codon. In these cases, 3′-UTR sequences were further extended until in-frame stop codons could be located for each sequence. Sequences with more than one consecutive *N* were considered to have a compromised reading frame, and so the poly *N* sequence and all 5′-sequence upstream in that frame was excluded; only edge gaps resulting from this procedure were permitted. Genes for which high-quality alignments could not be produced were excluded, reducing the total number of genes to 10,577. Exclusion occurred if the number of internal gaps plus ambiguous sequences (*N*) was more than 25% of the total number of character columns in the alignment. We then additionally excluded “transposable element genes” that slipped through the previous retrotransposon filter, as well as “merged ORFs” (i.e., now annotated as only part of a gene) bringing the total to 10,537.

### Identification of SCPs

If at least one strain lacked a stop codon that aligned with the annotated stop codon of the reference strain, that gene was flagged as containing a SCP. Presence of an aligned stop codon in all strains does not, however, rule out the presence of an earlier stop codon, nor the presence of an indel shifting the aligned stop codon out of frame. For each strain of a gene with aligned stop codons, we walked back one triplet at a time, looking for premature stop codons. A premature stop codon may either be the true, in-frame stop codon of the protein, or it may indicate a frameshift, leading to multiple premature stop codons that are overwhelmingly likely to be present out of frame. Either way, a premature stop codon caused the gene to be flagged as containing a SCP. A total of 4,147 genes were identified that had evidence of SCP. The walkback procedure continued until the annotated start codon was reached, clearly establishing frame. The start codon of the reference sequence was used to annotate protein lengths and other metrics of the protein.

An allele present only in a single strain is likely either to be a sequencing error, or to represent a deleterious mutation of little evolutionary interest. We therefore excluded singleton alleles, leaving 1,336 genes with nonsingleton evidence of SCP.

Nonsingleton SCP genes were excluded from further analysis if the outgroup was ambiguous with regard to inference of ancestral stop codon position. This is the case when the orthologous sister gene is also polymorphic in stop codon position, or when there is no data for the orthologous sister gene. These exclusions reduced the number of nonsingleton SCP genes in our analysis to 957.

**Fig. 1.—:**
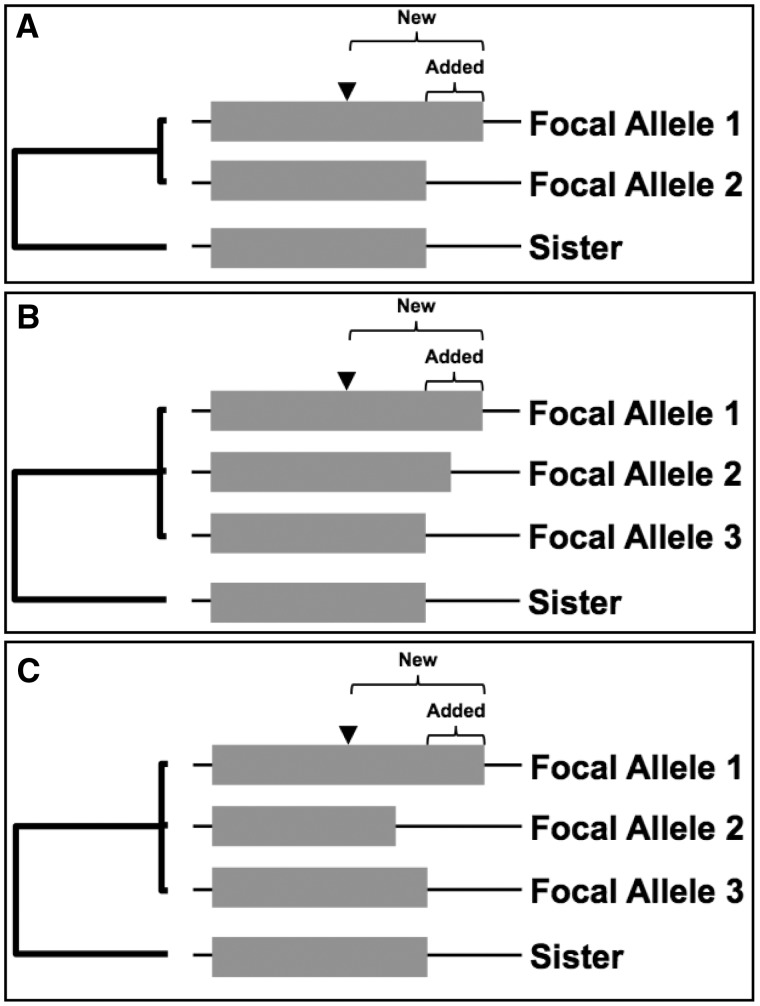
(*A*) The stop codon position in the sister species was used as an outgroup to determine whether a SCP was caused by an addition to or a subtraction from the ancestral coding sequence. Additions can result either from point mutations eliminating the stop codon or from indels that knock the stop codon out of frame. In the latter case, we distinguish between added amino acids that increase the total length of the protein, and new amino acids that include all novel amino acids following the indel. (*B*) When the SCP involves more than two stop codon positions, inference is more complicated. Here, at least one addition took place, plus one event that could have been either an addition or a subtraction. (*C*) At least one addition and one subtraction must have occurred to explain this phylogeny. More complex cases with more than 3 stop codon positions were classified using the same logic. While it is in principle possible to use the strain phylogeny (Liti et al. 2009) to distinguish the order of events in these cases, there is enough outcrossing between strains (Ruderfer et al. 2006) such that the gene tree may not match the strain tree, and so this was not done.

Nonsingleton SCP gene sequences were then realigned with their monomorphic sister reference sequences and reanalyzed for SCP. Genes were excluded if the stop codon position in the monomorphic sister species was not shared with any of the focal species alleles, reducing the number of genes to 817. The remaining genes were then classified as additions, subtractions, or ambiguous events (fig. 1). Alignments of genes that were classified as additions were manually checked for quality and poorly aligned sequences were removed. The remaining sequences were then realigned and edges were cleaned using the extend and prune algorithm described above.

Ribosomal footprinting data were used to confirm that putative C-terminal additions did in fact involve genuine protein-coding genes, since screening for C-terminal additions has the potential to enrich for “genes” whose annotation as protein-coding is false. We downloaded ribosomal footprint data and the corresponding transcriptomes (Ingolia et al. 2009) from the Gene Expression Omnibus (GEO) (http://www.ncbi.nlm.nih.gov/geo/) (GEO accession GSE13750). Transcript 5′- and 3′-boundary positions were obtained from table S4 of Nagalakshmi et al. (2008) and the positions of ORFs annotated as coding by SGD were noted within the context of the transcript. Plots of ribosomal density as a function of transcript position (supplementary fig. S1, Supplementary Material online) were manually examined for each putative C-terminal addition. In practice, manual annotation corresponded to discrimination according average ribosomal footprint density, calculated by dividing the number of hits to each nucleotide within the ORF by the mRNA concentration. Footprints were categorized into strong (>0.03), moderate (0.03–0.015), low (0.015–0.005), and no evidence (< 0.005), based on average ribosome density across the ORF region for each gene across both replicates of the data of Ingolia et al (2009).

## Results

We found 817 genes that had an SCP in one species, and where the sister species was monomorphic for the presumably ancestral allele. Six hundred sixty-one of these polymorphisms involved 2 nonsingleton stop codon position allelic variants, 120 involved 3, and 36 involved 4 or more. In 63 of these cases, the polymorphism appeared to involve an addition, that is, the conversion of 3′-UTR sequence into coding sequence. Each of these cases was manually inspected, and we were able to confirm good alignment between the 3′-UTR sequence in the ancestral allele and the C-terminal coding sequence in the addition allele, supporting the inference that the origin of the additional coding sequences was noncoding. However, two of the cases involved introns; due to their rarity in *Saccharomyces*, we used genomic data in our initial screening, and artifacts due to introns were eliminated at this late stage. This left 61 putative additions. Forty-six of these additions were straightforward inferences based on two alleles in the polymorphic species. When there were more alleles, additions could still be inferred, but details distinguishing the precise order of multiple events are less clear (fig. 1).

We performed two additional quality controls. First, we wanted to ensure that the additions we had found were to genuine protein-coding genes, rather than, for example, to pseudogenes under relaxed selection, which can easily lose and gain stop codons. We used ribosomal footprints (Ingolia et al. 2009) to assess the strength of evidence that genes annotated as protein-coding are in fact translated. Transcripts were available for 60 out of the 61 genes that had undergone putative additions. The gene that did not have transcript or footprint data available for analysis was YGL235W. YGL235W is annotated in SGD as a putative protein of unknown function, potentially a Cdc28p substrate; given the paucity of evidence, we excluded it from further analysis.

For the remaining 60 genes, we looked for evidence of translation via ribosomal binding to ORFs. We looked first under high stringency conditions for ribosomal binding evidence: a read sequence alignment quality score assigned by Ingolia et al. (2009) of 36 (the maximum possible), mapping to a unique genomic location that was at least 18 nt long (out a maximum possible of 32) (supplementary fig. S1, Supplementary Material online).

Under these high stringency conditions, 37 of the 60 genes transcribed in our data set showed strong evidence for translation, six genes showed moderate evidence, eight genes showed low evidence, and nine showed no evidence for translation. The 17 genes that contained low or no evidence were then examined under less stringent ribosomal footprint filters, allowing nonunique hits and an alignment score from 32 to 36 (supplementary fig. S2, Supplementary Material online). Taking these results into account, two genes moved from moderate to strong evidence (YDL056W, YLR095C), one gene (YOL100W) moved from low to moderate evidence, and two genes (YLR313C, YNL234W) moved up to low evidence for translation. These relaxed conditions did not support protein-coding capacity for the remaining 16 genes; for these 16, we conducted a literature search via SGD. Three of these 16 were annotated as “putative proteins” in SGD, whereas the other 13 genes showed evidence for the existence of a protein product using methods such as electrophoresis/chromatography separation followed by mass spectrometry, detection of tagged pulldowns in yeast expression systems, and/or documented catalytic activity of the purified form. Using this literature evidence, five low and seven no evidence genes were reannotated as having strong evidence for translation, whereas one low evidence gene was upgraded to moderate (ectopic expression of a His-tagged YNL234W within *Escherichia coli* rather than yeast). Some of these 13 genes, in particular those with high transcript levels, might be translationally regulated; this would explain their lack of ribosomal association in the profiling data despite evidence for protein presence in other studies. All five genes showing moderate evidence for translation based on riboprofiling were also upgraded based on strong literature evidence for protein translation. Table 1 annotates each addition gene based both on ribosomal profiling evidence for translation, and on total evidence for translation.

**Table 1:** Characteristics of All 55 Genes that Have Undergone Addition via Stop Codon Loss in Either *S. cerevisiae* or *S. paradoxus*, Including One Putative Gene Fusion

Systematic Name	Standard Name	Addition Species	Outgroup Allele Length (aa)	*“*Added” Sequence (aa)	“New” Sequence (aa)	Type of Addition	Number of Nonsingleton Alleles	Ribosomal Profile Evidence	Total Evidence	Gene Notes
YAL005C	SSA1	*S. cer*	643	18	49	Frameshift	2	Strong	Strong	ATPase activity
YBR014C	GRX7	*S. par*	204	15	22	Frameshift	3	Strong	Strong	Glutathione-disulfide reductase activity, involved in oxidative stress response
YBR046C	ZTA1	*S. par*	335	26	30	Frameshift	2	Strong	Strong	NADPH-dependent quinone reductase
YBR194W	AIM4	*S. par*	124	4	5	Frameshift	2	Moderate	Strong	Unknown functional activity; protein proposed to be associated with the nuclear pore complex
YBR264C	YPT10	*S. par*	200	10	15	Frameshift	2	Strong	Strong	Rab family, GTPase activity
YCR076C	FUB1	*S. cer*	243	11	22	Frameshift	2	Strong	Strong	Protein of unknown function; interacts with subunits of the 20 S proteasome
YDL027C	NA	*S. par*	421	3	4	Frameshift	2	Moderate	Strong	Protein of unknown function; nontagged protein is detected in highly purified mitochondria
YDL056W	MBP1	*S. par*	834	3	3	Point	3	Strong	Strong	DNA binding transcription factor activity; involved in regulation of transcription
YDL175C	AIR2	*S. cer*	343	3	15	Frameshift	2	Strong	Strong	RNA-binding activity
YDR062W	LCB2	*S. cer*	562	7	7	Frameshift	2	Strong	Strong	Serine C-palmitoyltransferase activity
YFR037C	RSC8	*S. par*	558	2	21	Frameshift	2	Strong	Strong	Component of the RSC chromatin remodeling complex involved in DNA binding activity
YGL058W	RAD6	*S. cer*	171	26	29	Frameshift	2	Strong	Strong	Ubiquitin-protein ligase activity
YGR004W	PEX31	*S. par*	463	1	1	Point	2	Moderate	Strong	Peroxisomal integral membrane protein; involved in negative regulation of peroxisome size
YGR059W	SPR3	*S. cer*	512	3	9	Frameshift	2	None	Strong	Structural septin protein activity; involved in sporulation
YGR136W	LSB1	*S. par*	242	19	48	Frameshift	2	Strong	Strong	Negative regulator of actin nucleation-promoting factor acitivity
YGR152C	RSR1	*S. par*	273	1	56	Frameshift	2	Strong	Strong	GTPase activty
YGR188C	BUB1	*S. par*	1,022	3	5	Frameshift	2	Low	Strong	Protein kinase activity; involved in cell-cycle checkpoint mechanism
YHR034C	PIH1	*S. cer*	341	1	1	Frameshift	3	Strong	Strong	Unknown functional activity; involved in RNA processing
YHR043C	DOG2	*S. cer*	247	5	7	Frameshift	3	Strong	Strong	2-deoxyglucose-6-phosphatase activity
YHR200W	RPN10	*S. cer*	268	18	24	Frameshift	2	Strong	Strong	Polyubiquitin binding activity
YHR206W	SKN7	*S. cer*	625	10	35	Frameshift	2	Strong	Strong	DNA binding transcription factor activity; oxidative stress response and osmoregulation
YIL110W	HPM1	*S. cer*	378	1	4	Frameshift	2	Strong	Strong	S-adenosylmethionine-dependent methyltransferase activity; ribosomal protein modification
YIL138C	TPM2	*S. cer*	162	3	45	Frameshift	2	Strong	Strong	Actin binding activity; involved in cell growth
YJL035C	TAD2	*S. par*	251	1	1	Point	2	Moderate	Strong	tRNA-specific adenosine deaminase activity; involved in t-RNA modification
YJL186W	MNN5	*S. cer*	587	21	22	Frameshift	2	Strong	Strong	Alpha-1,2-mannosyltransferase activity; involved in cell wall mannan biosynthesis
YJR075W	HOC1	*S. cer*	397	24	63	Frameshift	2	Strong	Strong	Alpha-1,6-mannosyltransferase activity; involved in cell wall mannan biosynthesis
YKL040C	NFU1	*S. par*	257	34	48	Frameshift	2	Strong	Strong	Involved in iron metabolism in mitochondria
YKL212W	SAC1	*S. cer*	624	5	7	Frameshift	2	Strong	Strong	Phosphatidylinositol phosphate phosphatase activity
YKR006C	MRPL13	*S. cer*	265	3	10	Frameshift	2	Strong	Strong	Mitochondrial ribosomal protein of the large subunit
YKR069W	MET1	*S. cer*	591	2	2	Point	2	Low	Strong	Uroporphyrinogen III transmethylase activity; sulfate assimilation and methionine biosynthesis
YLR095C	IOC2	*S. cer*	816	11	38	Frameshift	2	Strong	Strong	Nucleosome-stimulated ATPase activity; involved in chromatin remodeling
YLR142W	PUT1	*S. cer*	481	5	8	Frameshift	2	None	Strong	Proline oxidase activity; involved in utilization of proline as sole nitrogen source
YLR313C	SPH1	*S. cer*	650	13	13	Point	2	Low	Strong	Protein involved in shmoo formation and bipolar bud site selection
YLR318W	EST2	*S. cer*	877	2	5	Frameshift	4	None	Strong	Telomerase catalytic activity
YLR357W	RSC2	*S. par*	890	5	5	Point	3	Strong	Strong	ATP-dependent chromatin remodeling activity; part of the RSC chromatin remodeling complex
YLR359W	ADE13	*S. cer*	483	35	35	Frameshift	3	Strong	Strong	Adenylosuccinate lyase activity; involved in the nucleotide biosynthetic pathway
YLR407W	NA	*S. cer*	229	1	4	Frameshift	2	Strong	Strong	Putative protein of unknown function; null mutant displays elongated buds
YML047C	PRM6	*S. par*	353	3	6	Frameshift	2	None	Strong	Potassium ion transmembrane transporter activity; Pheromone-regulated protein
YMR011W	HXT2	*S. par*	542	6	15	Frameshift	2	Strong	Strong	High-affinity glucose transmembrane transporter activity
YMR240C	CUS1	*S. par*	437	28	41	Frameshift	2	Strong	Strong	Unknown function; required for assembly of U2 snRNP into the spliceosome
YNL234W	NA	*S. cer*	426	70	86	Frameshift	2	Low	Moderate	Protein of unknown function; may be involved in glucose signaling or metabolism
YNL251C	NRD1	*S. par*	576	15	21	Frameshift	3	Strong	Strong	RNA-binding protein activity; involved in the Nrd1 complex
YNL294C	RIM21	*S. par*	534	25	25	Frameshift	2	Low	Strong	pH sensor; involved in cell wall biosynthesis and alkaline pH response
YOL058W	ARG1	*S. cer*	420	714	722	Deletion	3	Strong	Strong	Arginosuccinate synthetase activity; involved in the arginine biosynthesis pathway
YOL086C	ADH1	*S. cer*	349	7	18	Frameshift	2	Strong	Strong	Alcohol dehydrogenase activity; involved with the reduction of acetaldehyde to ethanol
YOL100W	PKH2	*S. par*	1,082	1	10	Frameshift	3	Moderate	Strong	Serine/threonine protein kinase; involved in sphingolipid-mediated signaling pathway
YOR141C	ARP8	*S. par*	882	14	46	Frameshift	2	Strong	Strong	mRNA binding activity; involved in chromatin remodeling
YOR260W	GCD1	*S. cer*	579	11	52	Frameshift	2	Strong	Strong	Translation initiation factor activity; Gamma subunit of the translation initiation factor eIF2B
YOR387C	NA	*S. cer*	207	12	12	Point	3	None	Moderate	Unknown function; regulated by Aft1p transcription factor; highly inducible in zinc-depleted conditions
YPL183C	RTT10	*S. cer*	1,014	4	7	Frameshift	2	Strong	Strong	Cytoplasmic protein with a role in regulation of Ty1 transposition
YPL204W	HRR25	*S. cer*	494	51	66	Frameshift	2	Strong	Strong	Protein kinase activity; regulation of vesicular trafficking and DNA repair
YPL248C	GAL4	*S. par*	882	4	4	Point	2	None	Strong	DNA-binding transcription factor; involved in GAL genes activation in response to galactose
YPR068C	HOS1	*S. par*	471	5	5	Point	3	Low	Strong	Histone deacetylase activity
YPR113W	PIS1	*S. par*	221	59	61	Frameshift	2	Strong	Strong	Phosphatidylinositol synthase activity
YPR192W	AQY1	*S. cer*	306	22	34	Frameshift	4	None	Strong	Spore-specific water channel that mediates the transport of water across cell membranes

Note.—Evidence of translation and protein function is summarized; see main text for details.

The three genes with low total evidence (YIL152W, YML050W, and YNR034W-A) were excluded from further analysis as they demonstrated insufficient evidence for translation. This left 57 addition events for further analysis, including 44 confirmed by ribosomal footprinting, and 13 supported only by other literature.

Note that sequencing errors might occasionally lead to a false positive in the form of misannotation of an addition event when none took place. We deal with this primarily through the exclusion of addition allele singletons, a screening procedure that is also effective in excluding highly deleterious alleles, and for excluding mutations that occurred during the preparation of wild isolates for sequences. During a manual check of our 57 candidates, we found that two had been annotated as additions on the basis of two independent singleton mutations, each of which caused a frameshift that changed protein length by the same amount. We consider these double-singletons still to be singletons, and so in the interests of excluding all sequencing errors, we excluded these two genes from table 1 and from further analysis.

As yet another quality control against sequencing errors, we also looked at higher coverage resequencing data. Some *S. cerevisiae* and *S. paradoxus* yeast strains have since been resequenced at much higher coverage (Bergström et al. 2014). These data were accessed on September 9, 2013 from the Sanger Institute, and assemblies for 22 addition strains out of 75 were retrieved. No sequencing errors were revealed for the stop codon addition sites at this late stage.

**Fig. 2.—:**
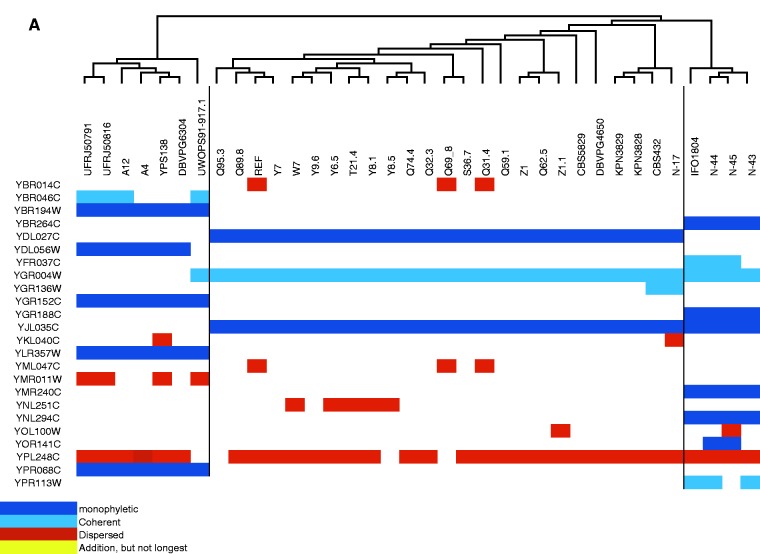

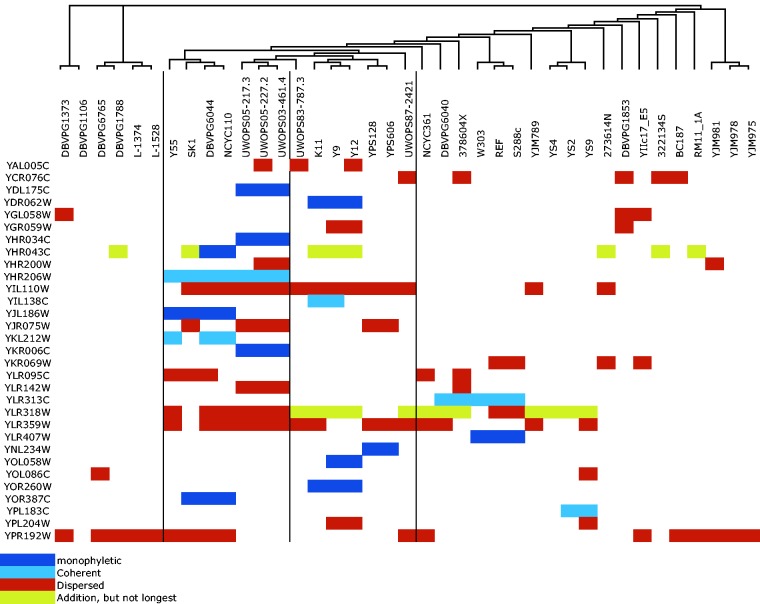
Distribution of addition allele across strains in *S. paradoxus* (*A*) and *S. cerevisiae* (*B*). Unrooted phylogenetic trees were taken from Liti et al. (2009). As is well-known, *S. paradoxus* shows more population structure (appearing here as dark blue monophyletic blocks or pale blue “coherent” near-monophyletic blocks) than *S. cerevisiae.* The strong phylogenetic pattern further demonstrates that these additions are not mere sequencing errors. A number of additions have risen to high frequency.

The distributions of additions across strains of *S. cerevisiae* and *S. paradoxus* are shown in figure 2. A significant number of addition alleles have risen to high frequency. Forty-eight percent (12 out of 25) addition alleles sit neatly on monophyletic clades within the tree of strains in *S. paradoxus* (Fig. 2*A*), whereas only 34% (11 out of 32) are found to be monophyletic in *S. cerevisiae* (fig. 2*B*). Those additions that are not monophyletic are widely dispersed across our sampled populations, especially in *S. cerevisiae* (fig. 2*B*). This is consistent with previous observations of greater population structure in *S. paradoxus* (Liti et al. 2009).

**Fig. 3.—:**
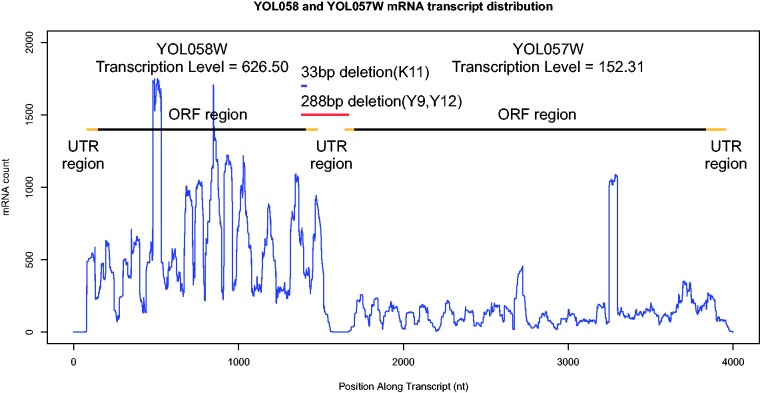
Transcription patterns do not support a gene fusion producing a single transcript. mRNA read counts from the Y12 strain *S. cerevisiae* (Skelly et al. 2013) were generated by sequence alignment using BFAST version 0.7a (Homer et al. 2009a, 2009b) and SAMtools version 0.1.19 (Li et al. 2009). ORF regions for YOL058W and YOL057W are shown by black bars. UTR regions (yellow bars) are based on the annotation of David et al. (2006) for the reference *S. cerevisiae* strain. The putative 288 bp deletion, which is expected to cause a fusion between two *S. cerevisiae* genes, is indicated by the red bar, whereas a smaller 33 bp deletion is indicated by the purple bar.

One extremely long addition (714 amino acids added to YOL058W) appeared to be the result of a 288 bp deletion that removed nine C-terminal amino acids, the ancestral stop codon and all of the 3′-UTR. The deletion ended in the 5′-UTR of the downstream gene (YOL057W) in-frame with its annotated start codon. Thus, the bulk of the addition consists of the 711 amino acid long ORF of YOL057W. Translation of this gene fusion can occur if the combined ORF is present on a single long transcript. The complete deletion of any transcription termination signal in the first gene’s 3′-UTR made continuous transcription a very real possibility. Because gene fusions are exceedingly rare in yeast (Durrens et al. 2008), we were surprised by this finding, and subjected it to a high level of scrutiny. The putative fusion is found in two closely related sake strains of *S. cerevisiae*, Y9 and Y12 (Liti et al. 2009). A third sake strain K11, the next closest relative to Y9 and Y12, has a 33 nt deletion in ARG1 that is a subset of the 288 bp deletion. This smaller deletion results in a premature stop codon causing an eight amino acid C-terminal deletion. To verify the existence of a full length transcript spanning the deletion region within these strains, we obtained Y12 strain RNA-Seq data from Skelly et al. (2013) and mapped it back to the Y12 *S. cerevisiae* genome. Upon visual examination, the deletion region had well-aligned reads flanking the deletion but lacked high quality reads that unambiguously spanned the deletion. We therefore next aligned the Y12 RNA-Seq reads to an alternative version of the Y12 genome assembly into which we reinserted the 288 bp deletion sequence. The new alignment (fig. 3) revealed strong hits to the previously annotated UTR portions (David et al. 2006) of the 288 bp deletion region. Figure 3 is compatible with two distinct genes, with different transcription levels, and is entirely inconsistent with a gene fusion. The annotation of a 288 nt deletion is clearly an error in both the Y12 and the Y9 genome assemblies. The 288 deletion is flanked by short poly-A sequences, which might be responsible for this replicated error.

Excluding the discredited gene fusion, the distribution of both “new” and “added” polypeptide lengths is shown in figure 4*A*–*C.* All novel amino acids created as a result of the SCP mutation are denoted as new, including those that are frameshifted prior to the ancestral stop codon, whereas added amino acids only include those novel amino acids that extend beyond the stop codon of the sister species allele. In other words, new amino acids include both alternative reading frames and 3′-UTR, whereas added includes only the latter (fig. 1). For comparison, we show the distribution of additions that would be to the next in-frame stop codon. Additions occurring during evolution are shorter than those expected from our control readthrough hypotheticals (fig. 4*D*; *P* = 0.035; two-tailed *t*-test on transformed data). This agrees with the expectation that longer additions to the gene are more likely to be deleterious than short ones, but the effect size is surprisingly modest (Added AA mean = 6.87; Readthrough AA mean = 9.75; fig. 4*D*). The shortest addition was 1 amino acid (YGL004W, YGR152C, YHR034C, YIL110W, YJL035C, YLR407W, and YOL100W), and the longest addition was 70 amino acids (YNL234W) (table 1). The smallest number of new amino acids was 1 (YGR004W, YHR034C, and YJL035C), and the largest number of new amino acids was 86 (YNL234W).

**Fig. 4.—:** The frequencies of C-terminal extension lengths per gene within *S. cerevisiae* and *S. paradoxus.* See figure 1 for the distinction between added (*A*) and new (*B*) amino acids. The “readthrough” histogram (*C*) is based on the number of amino acids that would be added to a gene if the stop codon were removed and translation were to read through to the next in-frame stop codon. Genes that did not reach a stop codon prior to the end of their UTR boundary as predicted by Nagalakshmi et al. (2008) were excluded. (*D*) The geometric mean and 95% confidence interval for added, new, and readthrough amino acid distributions. Data were approximately normal or truncated normal following a log transformation, so this transformation was used for statistics, with figure 4*D* generated through a back transformation. Added sequences are shorter than readthrough controls (*P* = 0.035; two-tailed *t*-test on transformed data). The still greater length of new amino acid sequences results from a statistical artifact; for many frameshifts that created smaller numbers of new amino acids, an early stop codon, earlier than the ancestral stop codon, would have prevented inclusion in our data set
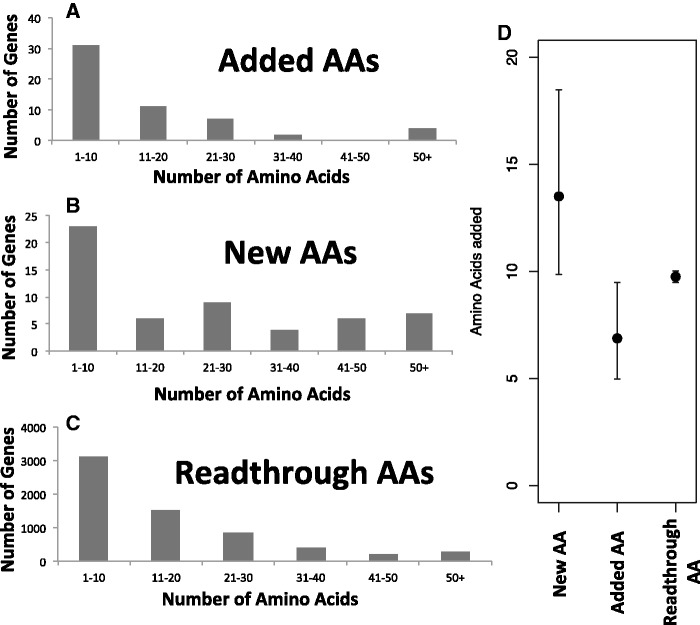
.

Of the 54 identified addition genes, 9 were caused by a point mutation that destroyed the stop codon and 45 were caused by a frameshift mutation upstream of the stop codon. Note that a high proportion of frameshifts relative to point stop codon losses is expected on the basis of a larger mutational target size, and indeed a ratio of 5:50 has previously been observed in fixed additions between mouse and rat (Giacomelli et al. 2007). However, a 19:20 ratio of in-frame:frameshifted additions was previously observed in fixed differences between yeast species, and this difference was attributed to the action of the yeast prion [PSI^+^] (Giacomelli et al. 2007). This bias toward in-frame additions is not reproduced in our data on the shorter timeframes corresponding to polymorphisms, where we have a ratio of 9:45 (*P* = 0.0009, G-test on contingency table), which is indistinguishable from the mouse:rat ratio (*P* = 0.2).

**Fig. 5.—:**
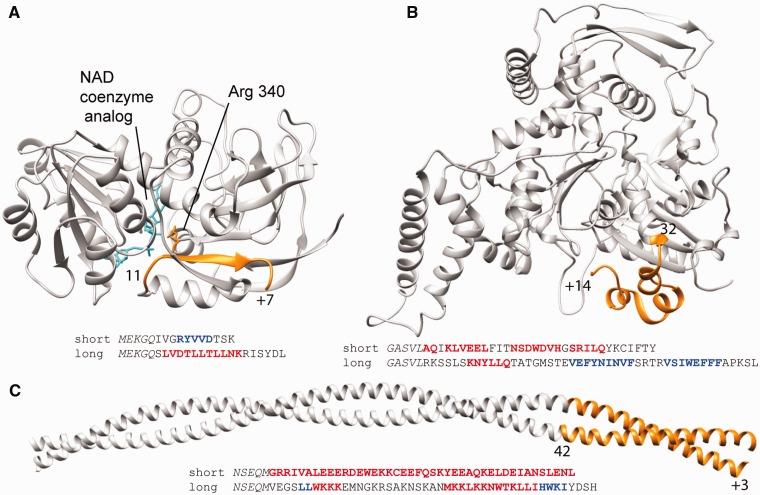
Three proteins with additions that may impact protein structure. (*A*) Alcohol dehydrogenase I from S. cerevisiae S288C, PDB ID 2HCY, chain A, residues 1–347, (*B*) Actin-related protein 8 from *S. cerevisiae* S288C, PDB ID 4AM6, chain A, residues 248-881, (*C*) Tropomyosin 2, homolog from *O. cuniculus* shown, PDB ID 2W49, chains A and B, residues 39–200. The ribbon diagram in each panel shows the portion of the protein altered by frameshift in orange, with the length of the altered region as well as the increase in sequence length indicated. Below each structure the C-terminal sequences of the reference strain and the longest version are shown, preceded by five residues of the unaltered region of sequence, shown in italics. Sequences are annotated with actual or predicted locations of α-helix (red) and β-strand (blue) secondary structures. These locations are inferred from the *S. cerevisiae* S288C or homologous structure in the case of the reference strain, or predicted by Jpred 3 in the case of the longest version.

Among our 54 addition alleles, we identified at least four cases in which the new amino acid sequence can be predicted to cause significant alteration and/or expansion of a protein domain structure. Three of these cases involve frameshift-mediated replacement of sequence integral to a protein domain structure, coupled with addition of varying amounts of sequence at the C terminus (fig. 5); a fourth case involves a pure addition with essentially no sequence replacement. In each of the four cases, secondary structure prediction using Jpred 3 (Cole et al. 2008) suggests the possibility of changes in secondary structure, either within the existing domain structure or as part of a C-terminal extension, or both. We discuss each case in more detail below.

YOL086C (ADH1) encodes alcohol dehydrogenase I, the constitutive enzyme primarily responsible for producing ethanol during yeast fermentation of glucose (de Smidt et al. 2008). In the *S. cerevisiae* reference strain and the *S. paradoxus* outgroup, AdhI is a 347 amino acid protein composed of tightly associated catalytic and coenzyme-binding domains that combine to span the entire sequence (Raj et al. 2014). Two other *S. cerevisiae* strains have a 7-residue addition with another 11 new amino acid residues created via frameshift.

The ADH1 frameshift replaces a β-strand that is conserved in all medium-chain ADH enzymes (Raj et al. 2014). This strand is part of the catalytic domain but also contacts NAD coenzyme via the side chain of Arg 340 (fig. 5*A*). Interestingly, the frameshifted version conserves several buried hydrophobic residues in this β-strand, and the added sequence has an alternating polar-nonpolar pattern that might contribute additional, amphipathic β-strand structure. Secondary structure prediction using JPred3, on the other hand, suggests a switch to helical structure in the long version, perhaps due to high leucine content. In addition, Arg 340 is converted to the smaller, oppositely charged Asp. In human ADH2, there is a common polymorphism in which this Arg residue is substituted by Cys, resulting in changes in coenzyme binding and enzyme kinetics (Burnell et al. 1989; Davis et al. 1996). Thus, ADH1 is a case where changes in protein function, and possibly structure, are likely.

YOR141C (ARP8) encodes an actin-related protein involved in chromatin remodeling in the nucleus. Arp8 is an essential component of the nucleosome-modifying complex INO80, and deletion of Arp8 results in defects in DNA repair and cell-cycle progression (van Attikum et al. 2004, 2007; Kawashima et al. 2007). Arp8 binds actin as well as histones, both of which are part of the INO80 complex (Shen et al. 2003; Fenn et al. 2011). Sequences of Arp8 from the *S. paradoxus* reference strain and the *S. cerevisiae* outgroup both contain 881 amino acid residues, of which the C-terminal domain containing the actin fold comprises residues 248-881 (fig. 5*B*) (Saravanan et al. 2012). Two other *S. paradoxus* strains have a 14-residue addition with another 32 new amino acid residues created via frameshift.

The frameshift replaces and expands the sequence of a small three-helix subdomain that is broadly conserved in actin and actin-related proteins (fig. 5*B*). JPred3 predicts conversion of helical secondary structure to β-strand as well as formation of β-strand structure in the added sequence. In actin itself, this C-terminal region directly participates in F-actin polymer formation (Holmes et al. 1990; Oda et al. 2009). Arp8 is related to actin, and binds actin, but does not polymerize or bind to the barbed ends of actin filaments (Fenn et al. 2011). Overall, the C-terminal three-helix subdomain plays no known role in the INO80 complex, so the functional consequences of disrupting its structure are hard to predict (Tosi et al. 2013).

YIL138C (TPM2) encodes a minor form of tropomyosin that interacts with actin filaments in cooperation with TPM1 to facilitate polar cell budding and growth (Drees et al. 1995; Pruyne and Bretscher 2000). Mutational analysis on TPM1 and TPM2 illustrated that TPM2 acts as a negative regulator of retrograde actin cable flow within yeast (Huckaba et al. 2006). Tropomyosins have an extremely simple coiled-coil structure (fig. 5*C***)** (Wu et al. 2010). Two strains of *S. cerevisiae* have a frameshift mutation that replaces the 42 C-terminal residues with a completely different 45-residue sequence.

The de novo sequence is likely to have less helical structure and other changes in its properties. Specifically, the COILS program (Lupas et al. 1991) indicates a high coiled-coil probability (0.7–0.9) for the 42 C-terminal residues in wild type, but a low probability for the de novo sequence (0.3–0.4). Secondary structure prediction with Jpred 3 also suggests loss of helix **(**fig. 5*C*). The net charge on the sequence is also converted from strongly negative to strongly positive. We conclude that the C-terminal structure is likely to be altered, though the implications for TPM2 function are not clear.

A fourth case of structural interest involves putative expansion of a helical transmembrane domain. YPR113W (PIS1) encodes a phosphatidylglycerophosphate synthase (COG0558), a group of transmembrane enzymes involved in lipid metabolism. Sequences of Pis1 from the *S. paradoxus* reference strain and the *S. cerevisiae* outgroup both contain 220 amino acid residues. Three strains of *S. paradoxus* have a frameshift mutation in Pis1 that replaces the C-terminal residue and adds 59 new residues.

Pis1 has no close homolog of known structure, but gives a weak BLAST hit (E∼0.027) to the CDP-OH phosphotransferase domain of IPCT-DIPPS from *Archaeoglobus fulgidus*, which contains approximately 200 residues and six transmembrane α-helices (PDB ID 4MND; not shown in figure 5 because the domain structure has no alignment overlap with the sequence introduced by the frameshift) (Nogly et al. 2014). Interestingly, the program TMHMM confidently predicts that the new sequence in the long version of Pis1 contains a transmembrane helix (posterior probability > 0.99 for residues 228–245) (Sonnhammer et al. 1998). Pis1 is thus a strong candidate for a pure evolutionary expansion of protein domain structure through C-terminal extension, potentially converting a six-helix into the seven-helix topology observed in many receptors.

### Comparison to Previously Published *Drosophila* Data

An interesting comparison study to ours is that by Lee and Reinhardt (2012) on polymorphisms in stop codon positions in *Drosophila melanogaster.* They reported 119 C-terminal extensions, all them involving a stop codon SNP rather than an upstream frameshifting indel, in addition to 438 premature stop codons. However, these numbers are based on annotating addition versus subtraction by assuming that whichever allele had the highest frequency was the ancestral form. Reasoning instead by parsimony with respect to an outgroup, and filtering to retain only those genes for which informative outgroup sequences were available, this data represents 106 C-terminal extensions. But if, as in the current study, we also exclude singleton addition alleles, on the grounds that they are likely to represent sequencing errors, strongly deleterious alleles, or recent mutations occurring in the laboratory during the few rounds of breeding used to construct a haploid or inbred genotype for *Drosophila* Population Genomics Project sequencing, we are left with only 50. We exclude four more because, while annotated as genes in the FlyBase version 5.15 used by Lee and Reinhardt (2012), they were no longer annotated as genes in FlyBase version 5.57. Four out of the remaining 46 annotated C-terminal extensions involved the same two genes, due to complications stemming from having more than two long alleles; excluding double counting brings the total number of C-terminal extensions down to 44. The “moderately supported” transcript of one remaining annotated gene was only approximately 198 nt long; its ORF was only 50 codons long and lacked other evidence for translation or function, and so we eliminated it. Finally, we eliminated 15 more genes because after performing reciprocal best hit retrieval of orthologs, we were unable to reproduce the SCPs reported by Lee and Reinhardt (2012). This might be a problem of unclear orthology, or alternatively of updated sequence data. Overall, these exclusions reduced Lee and Reinhardt’s reported number of C-terminal extensions from 119 to only 28 (listed in supplementary table S1, Supplementary Material online), illustrating the pitfalls of this kind of identification. Some of these 28 might still be artifacts related to splice annotation issues. Our reanalysis of this previous data set further illustrates the need for rigorous and conservative quality control measures in the annotation of rare evolutionary events.

## Discussion

Insight can be gained into the mysterious evolutionary conversion of noncoding sequences into coding by studying phylogenetically recent, rigorously vetted examples. De novo sequence evolution can be difficult to annotate. Loss of stop codons is one of the least ambiguous bioinformatics signals possible, where a mutation either directly removes the stop codon or causes a frameshift that bypasses the stop codon. The paucity of introns, let alone alternative splicing, in *Saccharomyces* makes these identifications even easier.

Any genome wide screen for large-scale evolutionary change will enrich for false positives. In other words, the presumably small number of falsely annotated genes in a well annotated model organism like *S. cerevisiae* are disproportionately likely to be picked up by screens such as ours. Out of 59 annotated cases with singletons and hence sequencing errors already excluded, 5 failed to meet our stringent screening criteria: the putative gene fusion because of a likely replicated assembly error, and 4 of the C-terminal additions because of insufficient evidence that the annotated genes truly were real genes, as demonstrated not only by “absence of evidence” considerations, but also via evidence of absence of translation (at least in the reference strain in rich media), as seen in ribosomal profiling data.

The false positive problem is particularly pronounced in the study of de novo genes. Because true de novo protein-coding genes are likely to be annotated, at best, as “putative proteins of unknown function,” the kind of quality controls performed here would exclude their study. In contrast, studying recent C-terminal extensions to well-annotated proteins allows us to have high confidence both that the sequence of interest is now truly protein-coding, and that it has recently arisen from a noncoding sequence.

Our study further highlights the severity of this false positive enrichment problem for rare evolutionary events. Our putative gene fusion is a cautionary tale about the quality of novel strain assemblies even in model organisms as well annotated as *S. cerevisiae.* Replication across the assemblies of two different strains was not enough to eliminate this error; we were only able to detect this assembly error as a result of a high coverage RNA-Seq data set. This calls into question the reliability of gene fusion identifications that rely on genomic data sets and/or a single assembly. Our quality filters also indicate the difficulties, even in the best-studied species, of reaching a “definitive” annotation of gene content. That said, ribosomal profiling holds promise as a technology for improving this annotation (Guttman et al. 2013; Ingolia et al. 2014), both by excluding genes, as done here, and also through discovering new ones too short and too little conserved to be annotated by other means (Wilson and Masel 2011; Smith et al. 2014).

Our study, using a set of stringent conditions, has identified 54 very recent instances of the conversion of noncoding 3′-UTR sequence into coding C-termini. In each instance, phylogenetic reconstruction using outgroup sequences supports annotation of the shorter allele as ancestral, and at least two yeast strains share the longer derived allele. We took pains to exclude sequencing errors and falsely annotated genes. Note that our exclusion of all singletons due to risk of sequencing errors may have caused us to also discard many true positives, especially in phylogenetically isolated strains. But the emphasis of a study of rare events must be on the exclusion of false positives.

C-terminal extensions are surprisingly well tolerated. For example, although the presence of the [*PSI*^+^] prion substantially increases stop codon readthrough at a large number of genes (Baudin-Baillieu et al. 2014), it is nevertheless found in some wild strains of yeast (Halfmann et al. 2012). As a second example, “programmed” stop codon readthrough has been reported in *Saccharomyces* (Namy et al. 2002, 2003; Artieri and Fraser 2013), mammals (Geller and Rich 1980; Yamaguchi et al. 2012; Eswarappa et al. 2014; Loughran et al. 2014; Stiebler et al. 2014), *Drosophila* (Xue and Cooley 1993; Klagges et al. 1996; Steneberg et al. 1998; Jungreis et al. 2011; Dunn et al. 2013), and other organisms (Jungreis et al. 2011; Freitag et al. 2012). None of our 54 stop codon loss events occurred in genes known to be subject to programmed stop codon readthrough. But the existence of programmed readthrough in other genes, as well as toleration of temporary increases in readthrough via [*PSI*^+^], make it less surprising that constitutive C-terminal extensions also occur during evolution. A number of fixed C-terminal extensions were already known (Giacomelli et al. 2007; Vakhrusheva et al. 2011). Here, we characterize newer events that have not yet become fixed within a species, generating the largest well-vetted set of such events to date.

Our study identified four interesting candidates (ADH1, ARP8, TPM2, and PIS1) for significant modification of a protein domain structure by introduction of new sequence. Though all of the genes identified with C-terminal extensions have important cellular functions, these four are particularly interesting due to the potential for the de novo sequence to replace one or more entire existing secondary structure elements and possibly add more at the C terminus. The actual impact of these sequence changes awaits experimental determination of structures for the extended versions. The only known structure for a completely de novo gene is an antifreeze protein (Chen et al. 1997; Nguyen et al. 2002), an intrinsically special case, and so the structural origins of novelty are a wide open question. In the study of C-terminal extension, these questions are accessible in a more contained form.

## Supplementary Material

Supplementary figures S1 and S2 and table S1 are available at *Genome Biology and Evolution* online (http://www.gbe.oxfordjournals.org/).
